# Supplementary material for: Comparable behaviour of ring and little fingers due to an artificial reduction in thumb contribution to hold objects
Source: PeerJ. 2020 Sep 17;8:e9962. doi: 10.7717/peerj.9962 (PMC7502246; doi:10.7717/peerj.9962)
Supplement: Supplemental Information 1 [file peerj-08-9962-s001.docx]

**Comparable behaviour of ring and little fingers due to an artificial reduction in thumb contribution to hold objects**

**Banuvathy Rajakumar^1^** and **Varadhan SKM^2^**

^1,2^Department of Applied Mechanics, Indian Institute of Technology Madras, Chennai, India.

**Corresponding Author:**

Dr Varadhan SKM

Assistant professor

Department of Applied Mechanics

Indian Institute of Technology Madras

Chennai - 600036

Tamil Nadu

+91-44-22574071

Email address: skm@iitm.ac.in

Corresponding Author ORCID: 10000-0002-5746-2340

**SUPPLEMENTARY INFORMATION**

**Supplementary Figure on the Average Normal and Tangential force during both conditions**

**
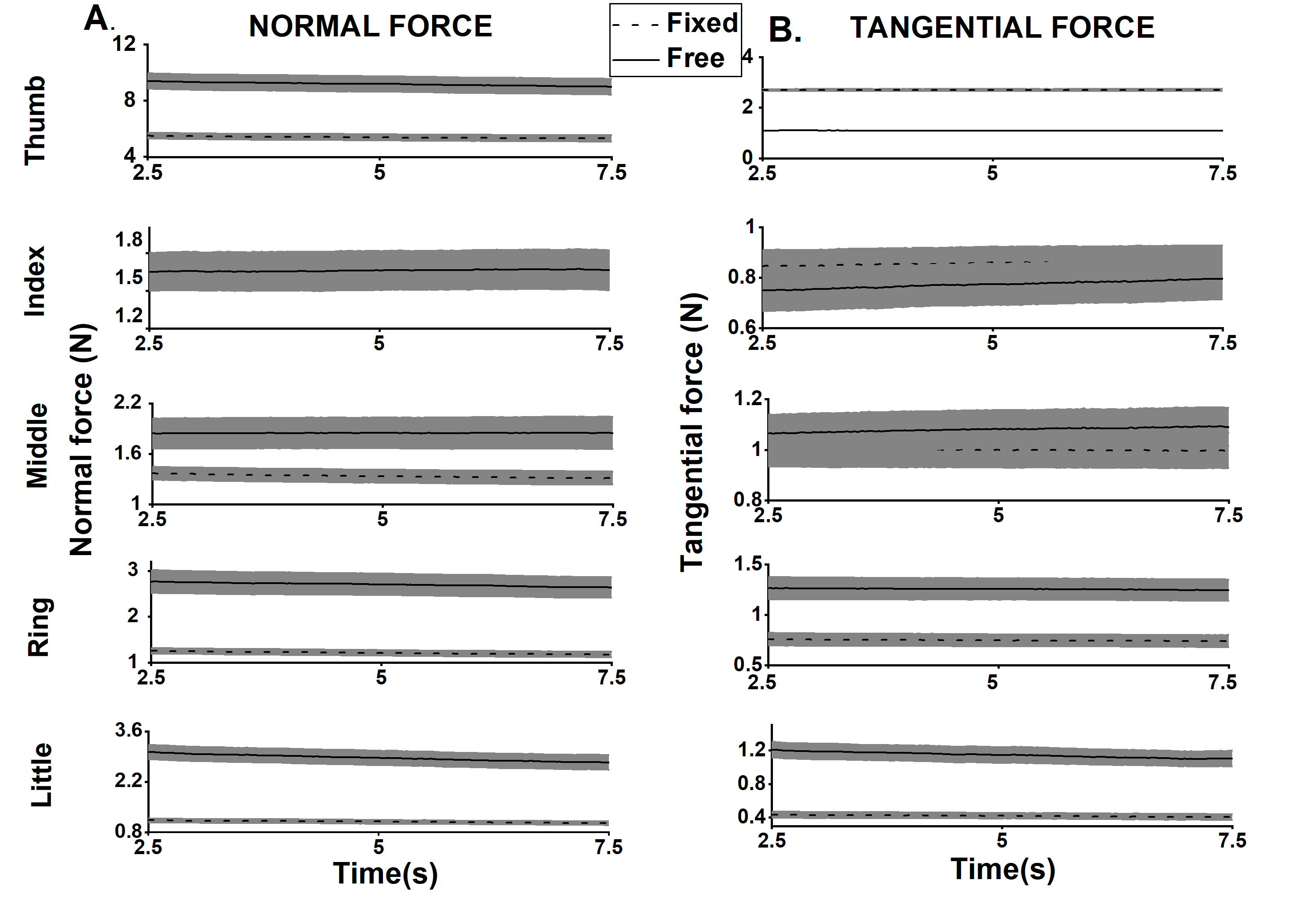
**

**Supplementary Figure S1. A. Average time profile of Normal force B. Average time profile of Tangential force of all the fingers during fixed and free conditions** Data shown are averages across subjects & trials in each condition. Fixed condition is represented with dashed lines and free condition represented with a solid line. Thick lines and shaded areas refer to the means and standard error of the means.

The mean (1.65N) and standard error of the mean (0.15N) of Index finger normal force during free condition found to be greater than the mean (1.63N) and standard error of the mean (0.11N) of Index finger normal force during fixed condition Hence, after plotting, line plot (mean) and the shaded portion (standard error of the mean) of the index finger normal force during free condition found embedded over the line plot and shaded portion of the index finger normal force during the fixed condition in Supplementary Figure S1 (A). Since the index finger normal force data (both mean and standard error) during fixed condition is found lower than the free condition, fixed condition data is found hidden under the free condition data when they are plotted. Similarly, the grey-shaded portion of the index and middle finger tangential force in free condition (i.e. standard error) hides the dashed line plot of the index and middle tangential force in the fixed condition at the midway.

**Supplementary Note on the Percentage change in Normal and Tangential forces**

Although some participants performed the “free” condition first, since we used fixed condition as control, we computed % change with respect to “fixed” condition regardless of the order in which the conditions were performed. The % Change in force was computed using the following equation.

$\% Change in Normal force (NF)= \frac{(Average NF in free condition-Average NF in fixed condition)}{Average NF in the fixed condition}*100$ (Supplementary Equation S1)

Likewise, % Change in tangential force was also computed.

**
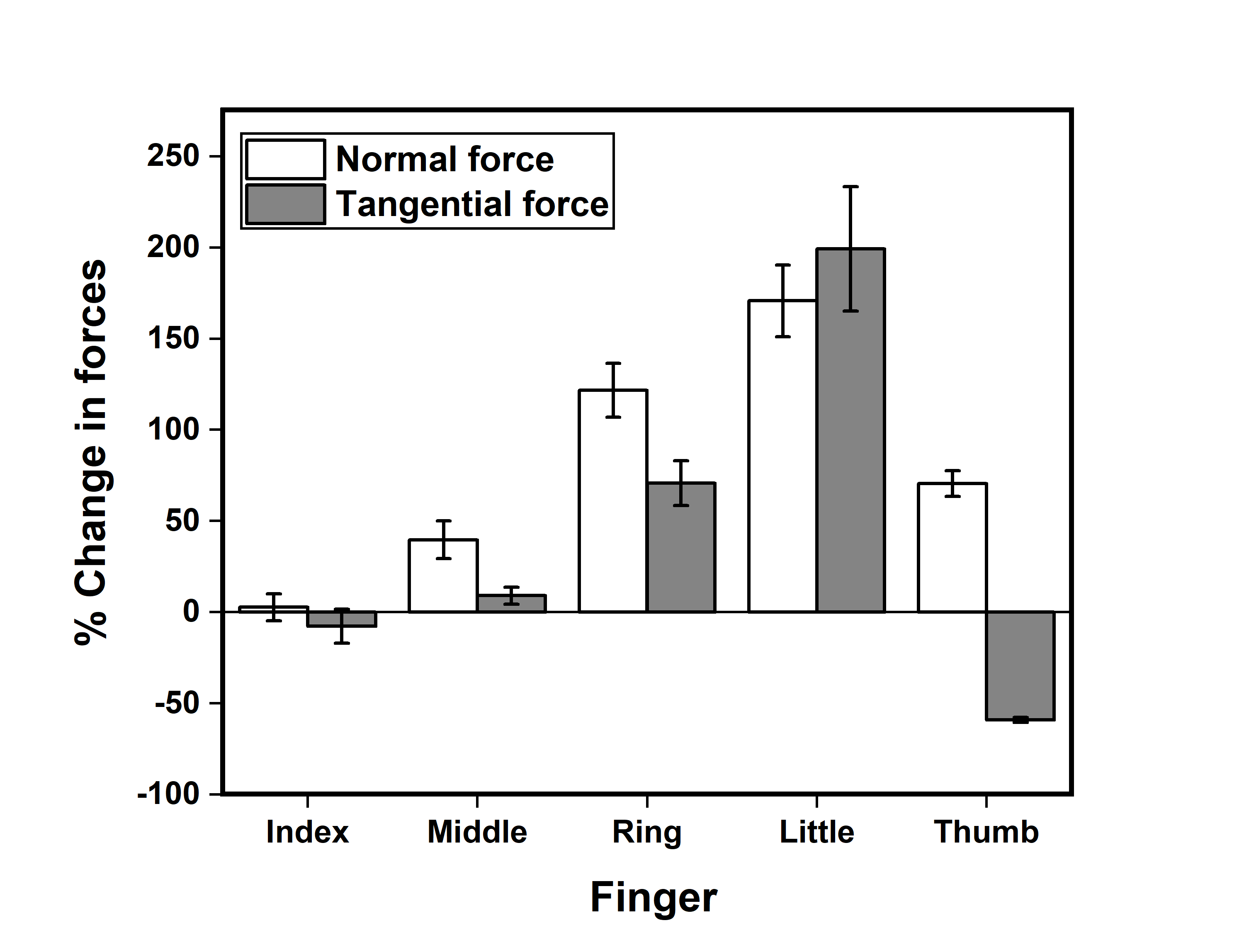
**

**Supplementary Figure S2.**  **Percentage change in the Normal and Tangential forces** Normal force is represented in white, and the Tangential force is represented in grey. All changes are computed for the free condition compared to the fixed condition. The normal force of the middle, ring, little and thumb increased by 40%, 121%, 170% and 70% respectively. The tangential force of the middle, ring and little fingers increased by 9%,70% and 199% respectively. The ring and little finger forces increased much more than the middle and index finger. Note that the thumb tangential force decreased by 60%, the normal force increased by around 70%.

We observed a 60% drop in the tangential force of the thumb. This drop was compensated by 70% and 199% rise in the tangential force of the ring and little finger in free condition with respect to fixed condition. In addition, there was a simultaneous increase in the normal force of the same fingers by 121 % and 170% respectively. The rise in the normal force of ulnar fingers was balanced by an increase in the normal force of the thumb by 70% (see Supplementary Figure S2).

**Supplementary Note on the Moment computation**

The unsteady thumb platform caused fingertip force re-distribution. Due to the re-distribution of fingertip forces, there was a change in the moment contribution of each finger. We computed moment due to virtual finger normal force $M_{n}^{\mathrm{VF}}$, the moment due to thumb tangential force $M_{t}^{\mathrm{Th}}$, the moment due to normal force of the ring ${(M}_{n}^{R})$ & the little ($M_{n}^{L})$ fingers, sum of the moment due to normal force of ring and little finger (${M_{n}^{R}+M}_{n}^{L}$) and total moment made by the virtual finger$M_{\mathrm{tot}}^{\mathrm{VF}}$ (see Supplementary Equation S6)

**Moments Computation:**

For an object in static equilibrium, we computed moments by various forces involved as described in previous studies (Zatsiorsky et al. 2003). Moment due to normal force of all fingers was calculated by,

$M_{n}^{j}=\left( d^{j}+COP_{y}^{j} \right)F_{n}^{j}$ (Supplementary Equation S2) $\mathrm{CO}P_{y}=\frac{M_{x}}{F_{n}}$ (Supplementary Equation S3)

Moment due to the normal force of the Virtual Finger was calculated by

$M_{n}^{I}+M_{n}^{M}+M_{n}^{R}+M_{n}^{L}=M_{n}^{\mathrm{VF}}$ (Supplementary Equation S4)

Moment due to thumb tangential force,

$M_{t}^{\mathrm{Th}}=-r^{\mathrm{Th}}F_{t}^{\mathrm{Th}}$ (Supplementary Equation S5)

Total Moment due to virtual finger is the sum of the Moment due to the normal and tangential force of virtual finger.

${M_{t}^{I}+M_{t}^{M}+M_{t}^{R}+M_{t}^{L}=M}_{t}^{\mathrm{VF}}$ (Supplementary Equation S6)

$M_{n}^{\mathrm{VF}}+M_{t}^{\mathrm{VF}}=Total Moment (M_{\mathrm{tot}}^{\mathrm{VF}})$ (Supplementary Equation S7)

j=Index(I), Middle(M), Ring(R), Little(L) or Thumb (Th). $\mathrm{CO}P_{y}$ refers to the center of pressure on the sensor surface about Y-axis (Zatsiorsky, Gao & Latash, 2003; Aoki et al., 2006). M_x_ refers to moment about the X-axis, n and t represent normal and tangential forces. d is the vertical distance from the geometric center of the handle to the center of a finger sensor. r refers to tangential moment arm (horizontal distance from the centre of the handle to the point of force application on the finger sensor).

In the fixed condition, d^Th^ is a constant. Hence, $M_{n}^{\mathrm{Th}}$ varies only due to changes in $F_{n}^{\mathrm{Th}}$. Note that in the case of free condition, both d^Th^ & $F_{n}^{\mathrm{Th}}$ are quantities that can vary at each point in time since the thumb sensor can be displaced vertically. The thumb tangential force is expected to produce a clockwise moment. The computed moments were averaged across the time, trials and participants.

**
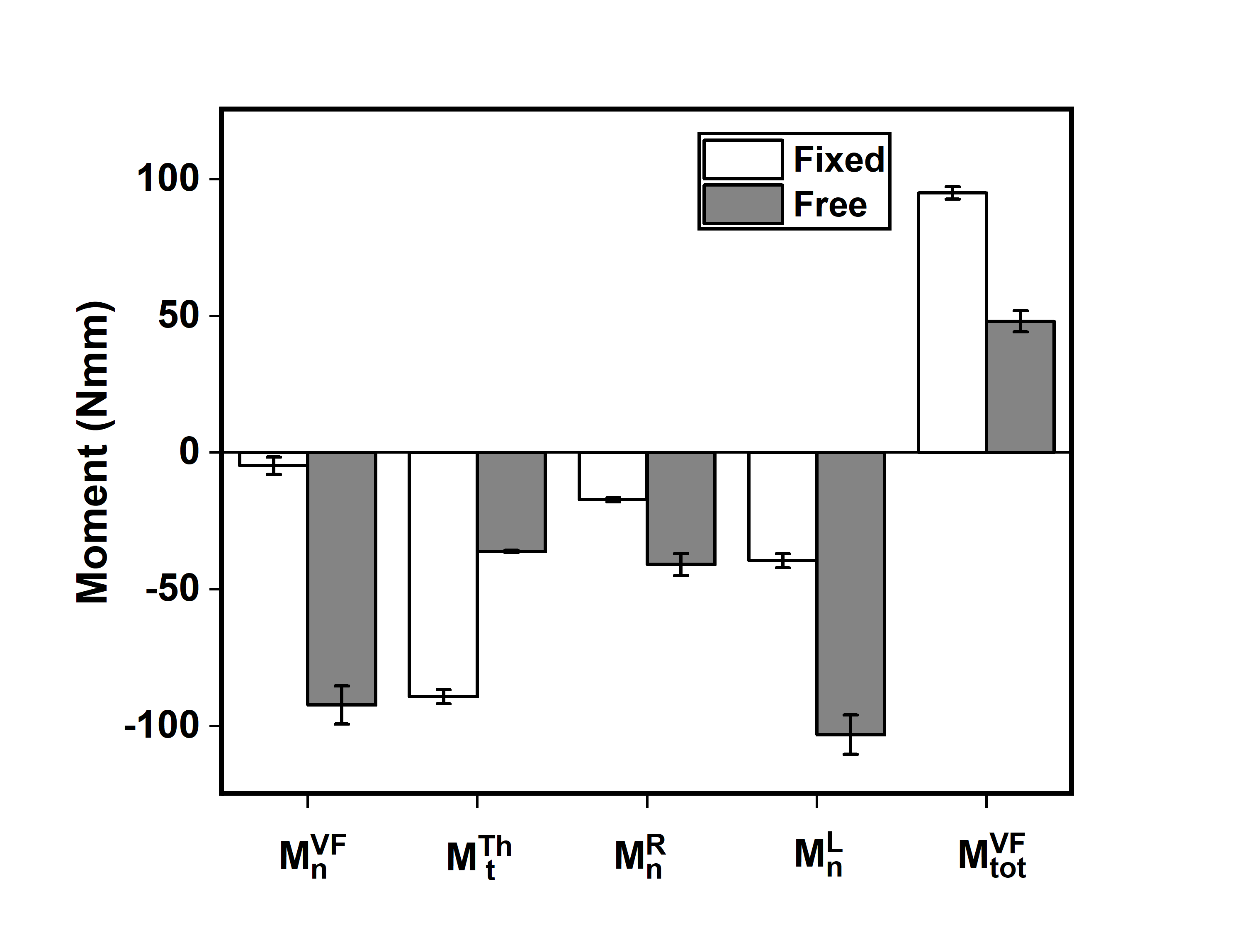
**

**Supplementary Figure S3.**  **Average Moment in fixed and free condition** Moment due to normal force of Virtual finger ($M_{n}^{\mathrm{VF}}$), Moment due to tangential force of thumb ($M_{t}^{\mathrm{Th}}$), Moment due to normal force of ring finger ($M_{n}^{R}$), Moment due to normal force of little finger ($M_{n}^{L}$) and Total moment due to Virtual finger ($M_{\mathrm{tot}}^{\mathrm{VF}}$) in fixed and free condition have been presented. In all cases, the moments between the two conditions are significantly different (p<0.001). Note the increase in $M_{n}^{\mathrm{VF}}$ (in the clockwise direction), the increase in $M_{n}^{R}$ , the increase in $M_{n}^{L}$ and the decrease in $M_{t}^{\mathrm{Th}}$. Also note the increase in total clockwise moment produced by virtual finger, approximately compensating for the decreased clockwise moment due to normal force of the thumb. The columns and error bars indicate means and standard error of the means.

The decrease in the clockwise moment of thumb tangential force $M_{t}^{\mathrm{Th}}$ was counteracted by the increase in the total clockwise moment (decrease in the counter-clockwise direction) due to the virtual finger$M_{\mathrm{tot}}^{\mathrm{VF}}$ (see Supplementary Figure S3). Increase in the moment due to normal force of virtual finger in free condition was mainly due to the increase in the normal force of middle, ring and little finger, not index finger.

We performed pairwise post-hoc Tukey tests on $M_{n}^{VF},$ $M_{n}^{R} and M_{n}^{L}$ which showed significant increase (p<0.001) in clockwise direction in free condition (-92.25 (VF), -40.89 (R), -103.15 (L)) Nmm compared to fixed condition (-4.79 (VF), -17.20 (R), -39.50 (L)) Nmm. Pairwise post-hoc comparisons showed a significant decrease (p<0.001) in the clockwise direction of $M_{t}^{Th}$ in free condition (-36.09 Nmm) compared to fixed condition (-89.24 Nmm). Statistically significant decrease in counter-clockwise direction was found in $M_{tot}^{VF}$ in free condition (48 Nmm) compared to the fixed condition (95.01 Nmm).

In fixed condition, the moment due to the normal force of VF was 4Nmm in the clockwise direction, but the same was much higher in the free condition. The increase in the clockwise moment due to normal force of VF (92Nmm) in the free condition was mainly due to the greater increase in the normal force of ring & little finger and a moderate increase in the normal force of the middle finger. In free condition, due to the thumb mobility, the moment due to thumb tangential force dropped across all participants as there was insufficient friction due to the slider mechanism found between the thumb sensor platform and the handle. This result is in agreement with the study of Aoki and colleagues (2006) where the participants exhibited a greater drop in the tangential force at the low friction contacts.

**Supplementary Note on the Synergy analysis**

Finger force covariation was quantified to examine the existence of synergy. For the purpose of this manuscript, we use a previous definition of “synergy” as “a neural organization of a set of elemental variables with a purpose of stabilizing a certain performance variable”(Latash, 2008).

As in the previous studies (Latash, 2008; SKM et al., 2012; Zhang et al., 2009; Sun, Zatsiorsky & Latash, 2011; Latash, Scholz & Schöner, 2002), synergy analysis on the mechanical variables was performed at two different levels: Virtual Finger-Thumb (VFTH) level and the Virtual finger (VF) level (see supplementary Note on Synergy analysis)

Index of synergy or index of covariation (∆V) was computed to quantify the amount of covariation that occurs within the elemental variables. Positive values of ∆V indicates negative covariation among the elemental variables. This, in turn, means the existence of synergy for that particular variable during the task. This index was computed across 30 trials for each participant separately, and then the average of ∆V (across time) was computed for each participant. This data was averaged across 15 participants, and SEM was found.

Synergy index was calculated by using the below equation.

$\Delta V=\frac{\sum Var(EVs)-Var(PV)}{\sum Var(EVs)}$ (Supplementary Equation S8)

EV refers to the elemental variables, and PV refers to performance variables. Index of covariation (∆V) was computed across 30 trials for each participant separately and then across time average of ∆V was performed for each participant. This data was averaged across 15 participants, and standard error of the mean was found. Fisher Z transformation was performed to the ∆V values of each participant for statistical analysis by using the following equation.

$\Delta V_{z}=0.5*ln\left( \frac{1+\Delta V}{1-\Delta V} \right)$ (Supplementary Equation S9)

Synergy index was calculated for the following performance variables found on the left-hand side of the below equations (Zhang et al., 2009).

**At VFTH level:**

$F_{n}^{\mathrm{VFTH}}=F_{n}^{\mathrm{VF}}+F_{n}^{\mathrm{Th}}$ (Supplementary Equation S10)

$F_{t}^{\mathrm{VFTH}}=F_{t}^{\mathrm{VF}}+F_{t}^{\mathrm{Th}}$ (Supplementary Equation S11)

$M_{\mathrm{tot}}^{\mathrm{VFTH}}=M_{n}^{\mathrm{VF}}+M_{t}^{\mathrm{Th}}+M_{t}^{\mathrm{VF}}+M_{n}^{\mathrm{Th}}$ (Supplementary Equation S12)

**At VF level:**

$F_{n}^{\mathrm{VF}}=F_{n}^{I}+F_{n}^{M}+F_{n}^{R}+F_{n}^{L}$ (Supplementary Equation S13)

$F_{t}^{\mathrm{VF}}=F_{t}^{I}+F_{t}^{M}+F_{t}^{R}+F_{t}^{L}$ (Supplementary Equation S14)

$M_{n}^{\mathrm{VF}}=M_{n}^{I}+M_{n}^{M}+M_{n}^{R}+M_{n}^{L}$ (Supplementary Equation S15)

$M_{\mathrm{tot}}^{\mathrm{VF}}=M_{n}^{\mathrm{VF}}+M_{t}^{\mathrm{VF}}$ (Supplementary Equation S16)

n and t stand for normal and tangential forces. I,M,R,L, Th and VF refers to Index, Middle, Ring, Little, Thumb and Virtual finger.

One-way repeated measures ANOVA were performed on the z-transformed synergy indices at VFTH & VF level with the condition as a factor.

For all the three performance variables at VFTH level, ∆V indices were positive during the fixed and free conditions (see Supplementary Figure S4). Note that the ∆V indices at VF level were positive for tangential force and total moment (M_tot_) in both conditions. Supplementary Figure S4 presents actual ∆V values, whereas statistical analysis was performed with Z-transformed ∆V values.

The observations about the synergy indices for the Z-transformed performance variables (∆V_z_ ) at VFTH and VF levels were tested by using one-way repeated measures ANOVAs with conditions as a factor. It was found that there was a significant difference (F_(1,14)_=8.9013;p<0.05, η^2^_p_ =0.38) between fixed and free condition on ∆V_z_ of the normal force at VFTH level. Also there was a significant decrease in ∆V_z_ of the tangential force (F_(1,14)_=58.88; p<0.001, η^2^_p_ =0.80) and ∆V_z_ of the total moment (F_(1,14)_=60.50; p<0.001, η^2^_p_ =0.81) in free condition (0.20, 0.23) when compared with the fixed condition (0.99, 1.09) at VFTH level. A significant increase (F_(1,14)_=8.23; p<0.05, η^2^_p_ =0.37) was seen in the ∆V_z_ of the tangential force (VF level) in free condition (1.08) in comparison to the same in fixed condition (0.71). ∆V_z_ of the total moment (VF level) showed a significant decrease (F_(1,14)_=57.99; p<0.001, η^2^_p_ =0.80) in free condition (0.73) compared to fixed condition (1.11).`


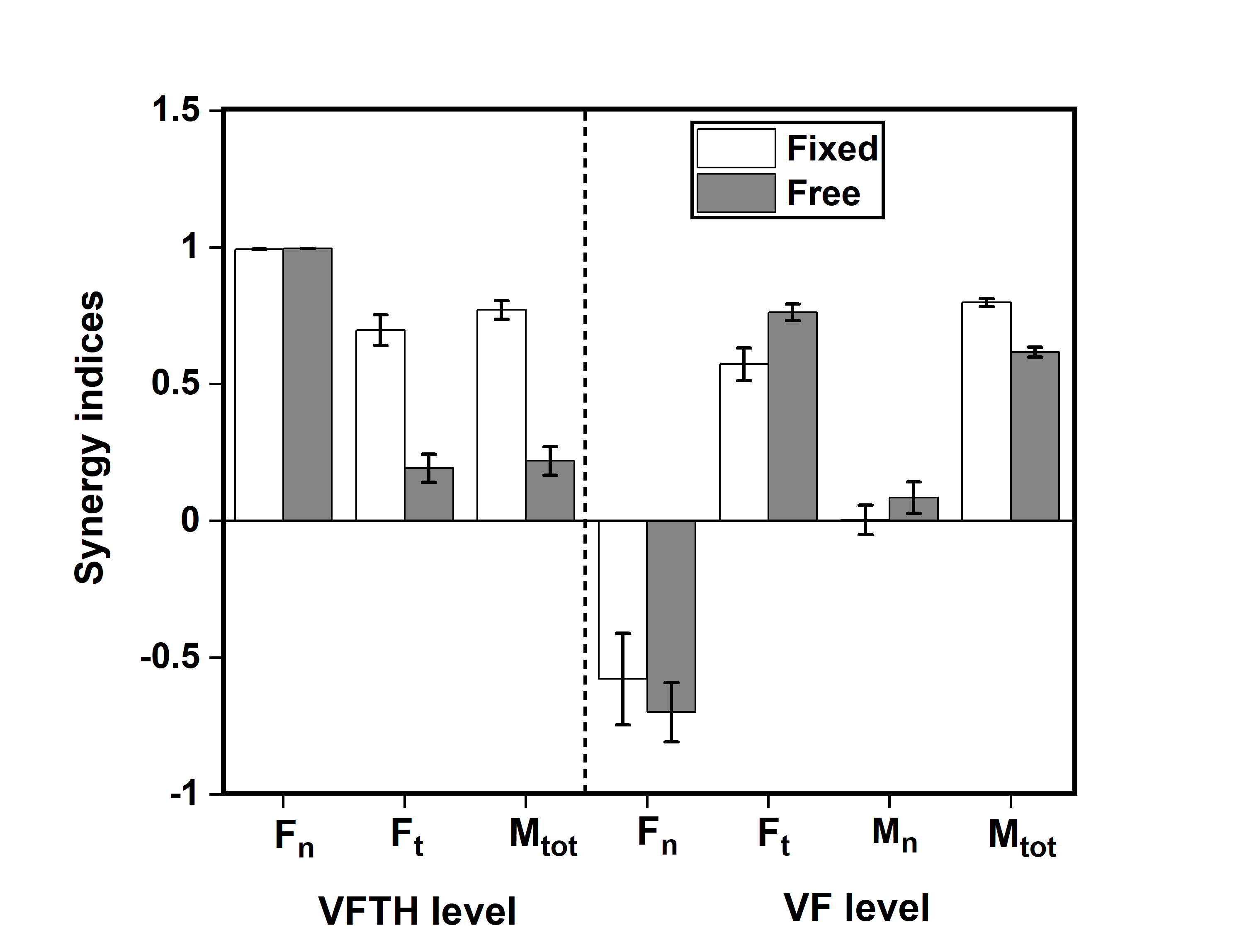


**Supplementary Figure S4 Synergy indices(∆V) for different performance variables at VFTH and VF level** Synergy index for the performance variables at VFTH level: Normal force(Fn), Tangential force(Ft) and Total moment (Mtot) are shown on the left side of the vertical dashed line. Synergy index for the performance variables at VF level: Normal force(Fn), Tangential force(Ft), Moment due to normal force(Mn) and Total moment (Mtot) are shown on right side of the vertical dash line. Synergy indices for Tangential force at VFTH level significantly decreased (p<0.001) in free condition compared to fixed condition Synergy indices for tangential force at VF level significantly increased (p<0.01) in free condition compared to fixed condition. Synergy indices for Mtot (VFTH and VF level) significantly decreased (p<0.001) in free condition compared to fixed condition. The columns and error bars indicate means and standard error of the means.

Prehension synergies have been explained within the framework of the Uncontrolled Manifold (UCM) hypothesis (Scholz & Schöner, 1999). Control of the hand and finger action has been viewed as a two-level hierarchical organization(MacKenzie & Iberall, 1994). The elemental variables at a higher level are forces and the moments produced by thumb and VF (VFTH level). At the lower level (VF Level), elemental variables are forces and moments generated by index, middle, ring, and little fingers. The sign and magnitude of synergy indices help to quantify the neural organization of the elemental variables involved in the task(Gelfand & Latash, 1998), as it is interpreted to be a direct measure of the CNS activity in response to any change in the task characteristics. Though this kind of co-variation analysis is different from the classical UCM analysis(Latash, Scholz & Schöner, 2002), the outcome measures in both approaches signify similar behaviour.

Positive index of covariation was observed for the performance variables like normal force, tangential force, and the total moment at VFTH level both during the fixed and free conditions (see Supplementary Figure S4). Large positive ∆V values (closer to +1) were found for the normal force at the VFTH level in both conditions suggesting the prevalence of a strong synergy. To achieve grasp stability, fingertip forces and moments (Mn and Mt) of VF and thumb adjust systematically among them, confirming the presence of synergy. In free condition, synergy indices for the tangential force and total moment in VFTH level decreased. This destabilization of tangential force and total moment in VFTH level could complicate the maintenance of rotational equilibrium during the free condition (when the thumb could move in the vertical direction). The drop in the ∆V value for the tangential force, the performance variable, signifies the reduction in synergic action when the handle equilibrium is disturbed. Meanwhile, for the other performance variable at the VFTH level, the normal force, ∆V value remains approximately the same. Thus, despite a decrease in the coordination of tangential force and total moment, the horizontal equilibrium is not compromised. At VF level, synergy index for tangential force increased in the free condition which means the facilitation of stronger synergies by the system. Negative index of co-variation was seen in the normal force in fixed as well as free condition. There was no synergy for the moment due to normal force at the VF level.

From Supplementary Figure S4, we observe that the synergy indices of performance variables like tangential force and total moment were different in free condition compared to the fixed condition. This reflects the fact that a synergic solution is preferred when the task characteristic is altered. There was a deterioration in the synergy indices of tangential force (a drop of 72%) at the VFTH (higher) level in free condition. However, there was a substantial increase of 33% in the synergy indices of tangential force at the VF (lower) level. This implies that the elemental variables involved in stabilizing tangential force at the lower level (i.e., tangential forces of index, middle, ring and little finger) co-adjust among themselves actively, thereby ensuring proper coordination within the individual fingers other than the thumb. An increase in the tangential force coordination at the lower level of the hierarchy compensated for the poorer tangential force coordination at a higher level. In free condition, synergy indices of the total moment at higher level dropped to about 71%, whereas at a lower level the drop was only 22%. Though there was a drop in both levels, the drops are not comparable. Such an inherent trade-off between synergies at two levels of a hypothetical hierarchy observed in our study are in broad agreement with previous studies on multi-digit synergies in similar prehension task (Gorniak, Zatsiorsky & Latash, 2007, 2009; Latash, 2008). We believe that the CNS preferred to change the strength of synergy for multiple performance variables (further with changes in multiple elemental variables at multiple levels of hierarchy) to achieve stability in task performance.

**Supplementary Note on the Linear discriminant analysis**

To examine how the change in tangential force produced by the thumb affected the moments produced by normal forces and hence, the static equilibrium of the object, we used a Linear Discriminant Analysis (LDA). LDA was performed separately between the following pairs of variables:

$F_{t}^{\mathrm{Th}} \& M_{n}^{\mathrm{VF}}$

$$F_{t}^{\mathrm{Th}} \& M_{n}^{R}$$

$$F_{t}^{\mathrm{Th}} \& M_{n}^{L}$$

$$F_{t}^{\mathrm{Th}} \& \left( M_{n}^{R}+M_{n}^{L} \right).$$

A linear discriminant classifier was trained with the set of data points on thumb tangential force and the moments mentioned above for the two conditions. We performed this analysis for the four pairs of variables mentioned above (see supplementary Figure S5, Figure S6, Figure S7, Figure S8).

**Classification accuracy**

We investigated the change in the moment caused by normal force due to the change in the tangential force of the thumb during the fixed and free conditions using linear discriminant analysis (LDA). The change in thumb tangential force resulted in a change in the normal force of the other fingers which in turn caused a change in the moment due to the normal force (see supplementary note on the moment computation). The two different conditions: fixed and free were considered to be the two different classes for the purpose of LDA. Both the classes were found to be linearly separable by a decision boundary that was constructed using LDA. The classifier was trained with 405 data points on thumb tangential force and moment due to normal forces of the individual fingers. LDA was able to predict the test data at an accuracy of 98%, sensitivity of 100%, specificity and precision of 97%, false-positive rate of 2% for all four cases. This result is illustrated in Supplementary Figure S5. The results for the other pairs of variables are included in the supplementary Figure S6, Figure S7 and Figure S8.

In free condition, data is spread wider across various values of moments due to variations in the normal force of virtual finger (see supplementary Figure S5). Though there was a constant moment arm of normal force for virtual finger in the free condition, moments varied widely because of the change in the normal force of ring and little finger. Moment due to normal force of little finger was greater than the moment due to normal force of ring finger as the little finger (‘peripheral finger’) is among the moment generating fingers (Zatsiorsky, Gregory & Latash, 2002). In the fixed condition, the thumb’s tangential force was spread in the shape of a vertical ellipse (in supplementary Figure S5). The tangential force of the thumb was found to be denser at approximately 2.7N and sparsely distributed between 2N to 3.5N. Moment due to normal force of VF was denser around 0Nmm. But the moment due to the normal force of ring and little fingers as well as the sum of the ring & little fingers was negative suggestive of a ‘clockwise moment’.


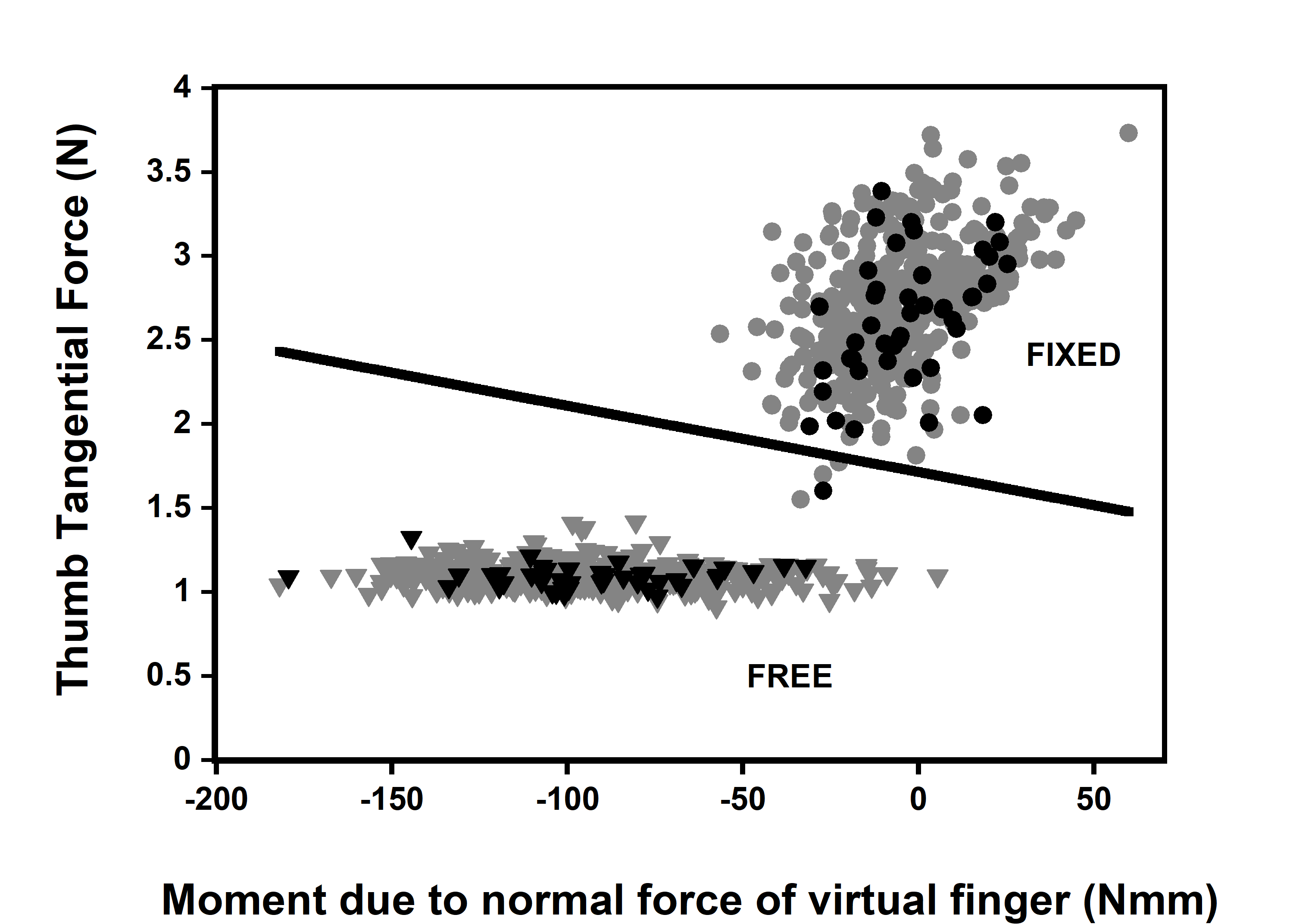


**Supplementary Figure S5.**  **Thumb tangential force (**$\boldsymbol{F}_{\boldsymbol{t}}^{\boldsymbol{Th}}$**) as a function of Moment due to normal force of Virtual Finger (**$\boldsymbol{M}_{\boldsymbol{n}}^{\boldsymbol{VF}}$**)** Each datapoint in the scatter plot represents time average of a single trial of a single subject. Data from all subjects are presented. Inverted triangle in grey & black colours are train (405) and test (45) datapoints in free condition. Circles in grey & black colours are train (405) and test (45) datapoints in fixed condition. Note: Only one test datapoint belonging to fixed condition was wrongly classified as free condition, so the accuracy of classification is 98%, sensitivity is 100%, specificity and precision is 97%, false positive rate is 2%.

**
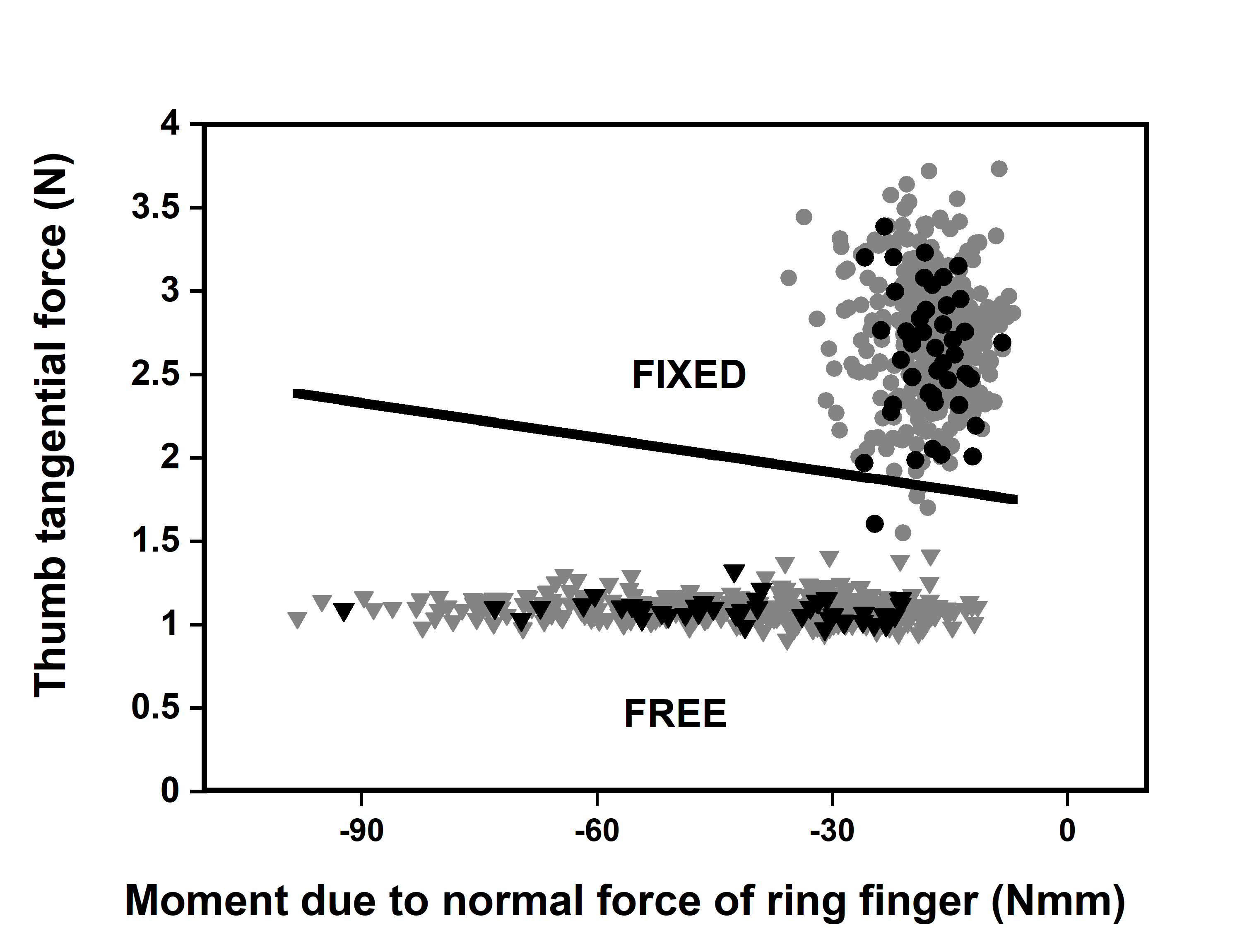
**

**Supplementary Figure S6.**  **Thumb load force (LF-TH) as a function of Moment due to the normal force of ring finger (Mn-R).** Each datapoint in the scatter plot represents time average of a single trial of a single subject. Data from all subjects are presented. Inverted triangle in grey & black colours are train (405) and test (45) datapoints in free condition. Circles in grey & black colours are train (405) and test (45) datapoints in fixed condition. Note: Only one test datapoint belonging to fixed condition was wrongly classified as free condition, so the accuracy of classification is 98%, sensitivity is 100%, specificity and precision is 97%, false positive rate is 2% in all the four cases.

**
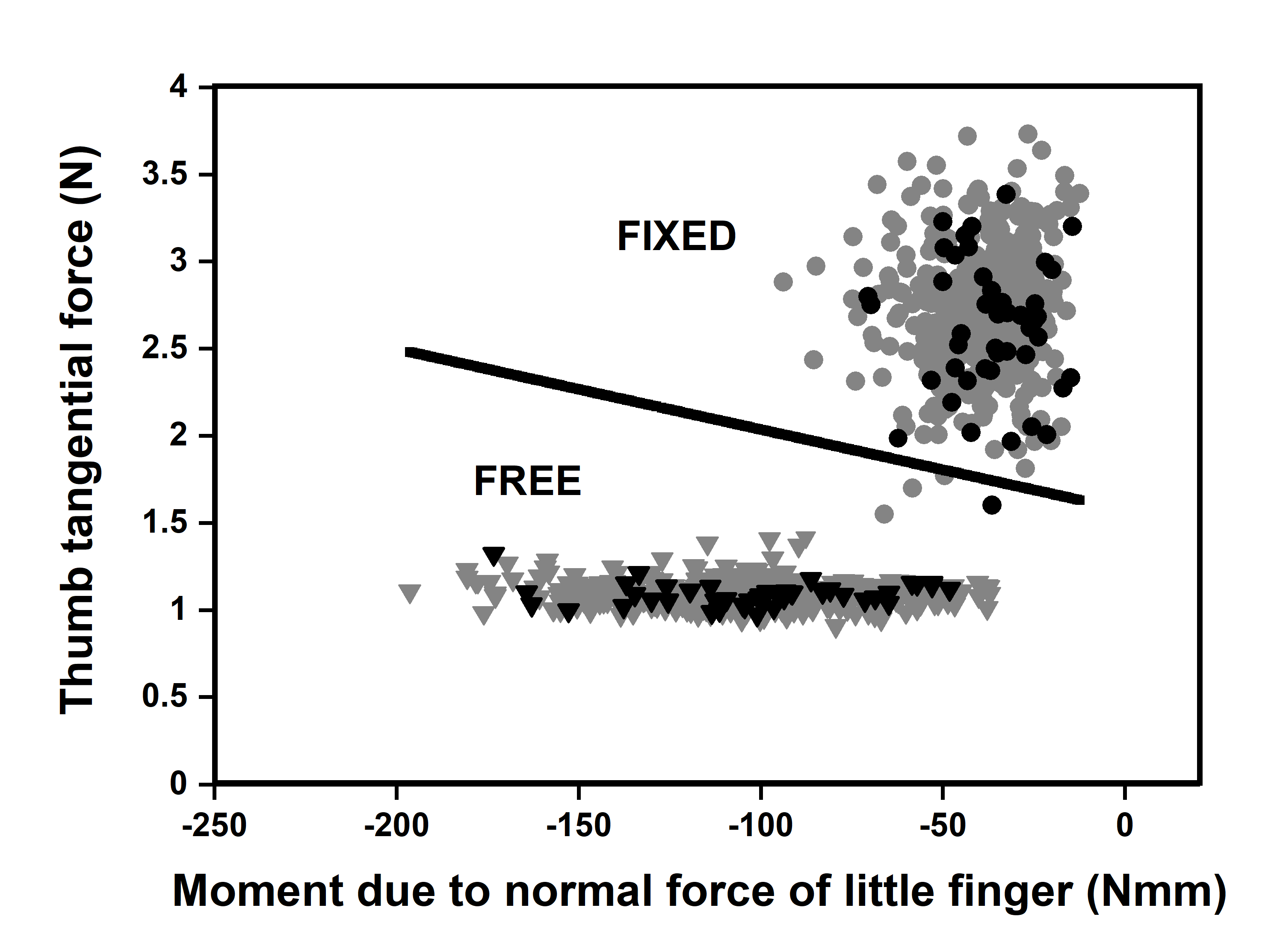
**

**Supplementary Figure S7.**  **Thumb load force (LF-TH) as a function of Moment due to the normal force of little finger (Mn-L).** Each datapoint in the scatter plot represents the time average of a single trial of a single subject. Data from all subjects are presented. Inverted triangle in grey & black colours are train (405) and test (45) datapoints in the free condition. Circles in grey & black colours are train (405) and test (45) datapoints in the fixed condition. Note: Only one test datapoint belonging to the fixed condition was wrongly classified as free condition, so the accuracy of classification is 98%, sensitivity is 100%, specificity and precision is 97%, the false-positive rate is 2% in all the four cases.


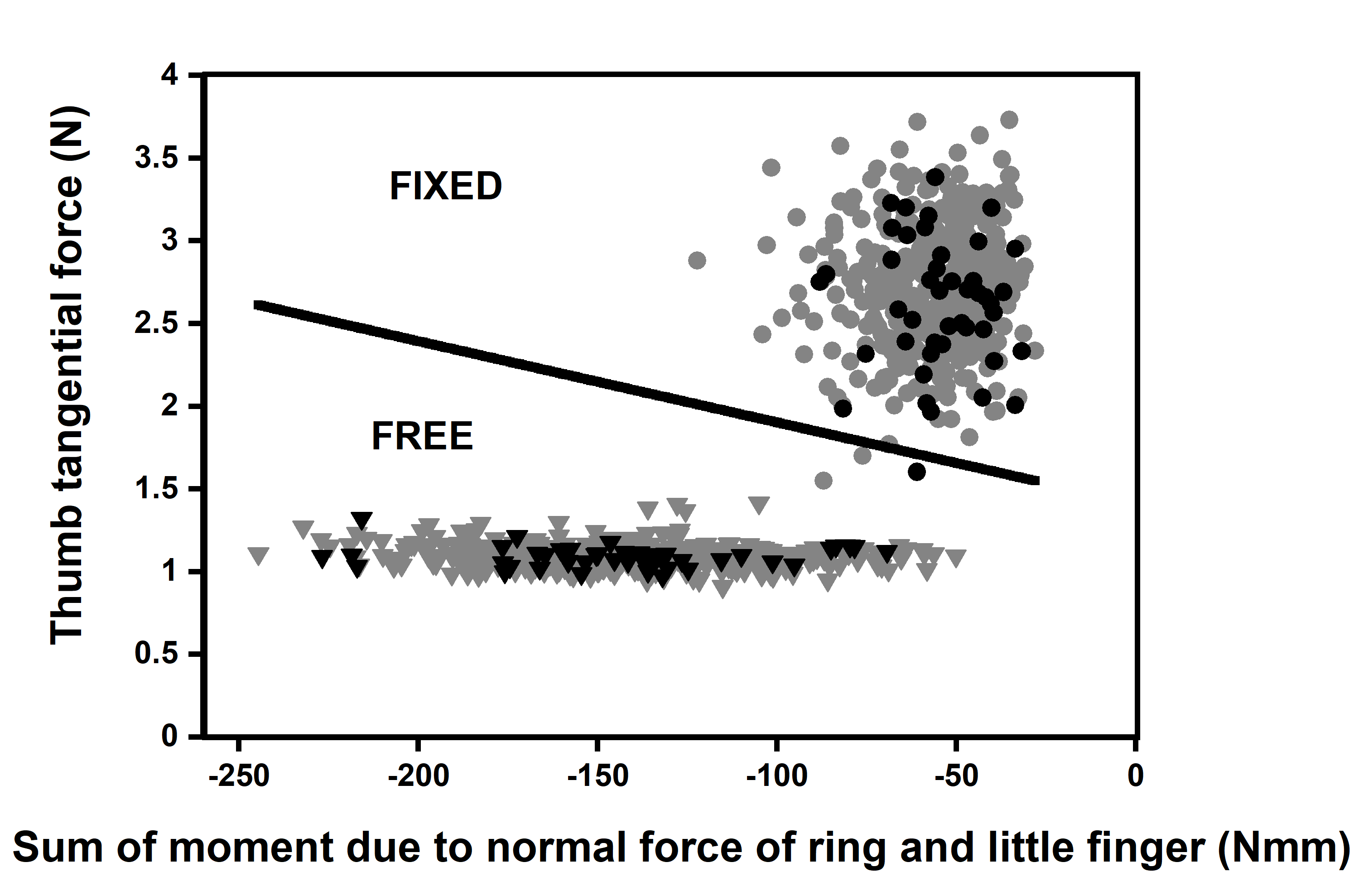


**Supplementary Figure S8.**   **Thumb load force (LF-TH) as a function of sum of the Moment due to normal force of ring and little finger (Mn-RL).** Each datapoint in the scatter plot represents the time average of a single trial of a single subject. Data from all subjects are presented. Inverted triangle in grey & black colours are train (405) and test (45) datapoints in free condition. Circles in grey & black colours are train (405) and test (45) datapoints in the fixed condition. Note: Only one test datapoint belonging to the fixed condition was wrongly classified as free condition, so the accuracy of classification is 98%, sensitivity is 100%, specificity and precision is 97%, the false-positive rate is 2% in all the four cases.

**References**

1. Aoki T, Niu X, Latash ML, Zatsiorsky VM. 2006. Effects of friction at the digit-object interface on the digit forces in multi-finger prehension. *Experimental brain research. Experimentelle Hirnforschung. Experimentation cerebrale* 172:425–438. DOI: [10.1007/s00221-006-0350-9](https://doi.org/10.1007/s00221-006-0350-9).
2. Gelfand IM, Latash ML. 1998. On the Problem of Adequate Language in Motor Control. *Motor Control* 2:306–313. DOI: [10.1123/mcj.2.4.306](https://doi.org/10.1123/mcj.2.4.306).
3. Gorniak SL, Zatsiorsky VM, Latash ML. 2007. Emerging and disappearing synergies in a hierarchically controlled system. *Experimental Brain Research* 183:259–270. DOI: [10.1007/s00221-007-1042-9](https://doi.org/10.1007/s00221-007-1042-9).
4. Gorniak SL, Zatsiorsky VM, Latash ML. 2009. Hierarchical control of static prehension: II. Multi-digit synergies. *Experimental Brain Research* 194:1–15. DOI: [10.1007/s00221-008-1663-7](https://doi.org/10.1007/s00221-008-1663-7).
5. Latash ML. 2008. *Synergy*. Oxford University Press.
6. Latash M, Scholz J, Schöner G. 2002. Motor Control Strategies Revealed in the Structure of Motor Variability. *Exercise and Sport Sciences Reviews* 30:26–31.
7. Li Z-M, Latash ML, Newell KM, Zatsiorsky VM. 1998. Motor redundancy during maximal voluntary contraction in four-finger tasks. *Experimental Brain Research* 122:71–78. DOI: [10.1007/s002210050492](https://doi.org/10.1007/s002210050492).
8. MacKenzie CL, Iberall T. 1994. *The Grasping Hand*. Elsevier.
9. Scholz JP, Schöner G. 1999. The uncontrolled manifold concept: identifying control variables for a functional task. *Experimental Brain Research* 126:289–306. DOI: [10.1007/s002210050738](https://doi.org/10.1007/s002210050738).
10. Sun Y, Zatsiorsky VM, Latash ML. 2011. Prehension of Half-Full and Half-Empty Glasses: Time and History Effects on Multi-Digit Coordination. *Experimental brain research. Experimentelle Hirnforschung. Experimentation cerebrale* 209:571–585. DOI: [10.1007/s00221-011-2590-6](https://doi.org/10.1007/s00221-011-2590-6).
11. Zatsiorsky VM, Gregory RW, Latash ML. 2002. Force and torque production in static multifinger prehension: biomechanics and control. I. Biomechanics. *Biological cybernetics* 87:50. DOI: [10.1007/s00422-002-0321-6](https://doi.org/10.1007/s00422-002-0321-6).
12. Zatsiorsky VM, Gao F, Latash ML. 2003. Finger force vectors in multi-finger prehension. *Journal of biomechanics* 36:1745–1749.
13. Zhang W, Olafsdottir HB, Zatsiorsky VM, Latash ML. 2009. Mechanical Analysis and Hierarchies of Multi-digit Synergies during Accurate Object Rotation. *Motor control* 13:251–279.
